# A novel combretastatin A-4 derivative, AC7700, strongly stanches tumour blood flow and inhibits growth of tumours developing in various tissues and organs

**DOI:** 10.1038/sj.bjc.6600296

**Published:** 2002-05-03

**Authors:** K Hori, S Saito, K Kubota

**Affiliations:** Department of Vascular Biology, Division of Cancer Control, Institute of Development, Aging and Cancer, Tohoku University, 4-1 Seiryomachi, Aoba-ku, Sendai 980-8575, Japan; Department of Nuclear Medicine and Radiology, Institute of Development, Aging and Cancer, Tohoku University, 4-1 Seiryomachi, Aoba-ku, Sendai 980-8575, Japan

**Keywords:** combretastatin A-4, tumour blood flow, tumour vessel, microcirculation, metastasis

## Abstract

In a previous study, we used subcutaneous LY80 tumours (a subline of Yoshida sarcoma), Sato lung carcinoma, and methylcholanthrene-induced primary tumours, to demonstrate that a novel water-soluble combretastatin A-4 derivative, AC7700, abruptly and irreversibly stopped tumour blood flow. As a result of this interrupted supply of nutrients, extensive necrosis was induced within the tumour. In the present study, we investigated whether AC7700 acts in the same way against solid tumours growing in the liver, stomach, kidney, muscle, and lymph nodes. Tumour blood flow and the change in tumour blood flow induced by AC7700 were measured by the hydrogen clearance method. In a model of cancer chemotherapy against metastases, LY80 cells (2×10^6^) were injected into the lateral tail vein, and AC7700 at 10 mg kg^−1^ was injected i.v. five times at intervals of 2 days, starting on day 7 after tumour cell injection. The number and size of tumours were compared with those in the control group. The change in tumour blood flow and the therapeutic effect of AC7700 on microtumours were observed directly by using Sato lung carcinoma implanted in a rat transparent chamber. AC7700 caused a marked decrease in the tumour blood flow of all LY80 tumours developing in various tissues and organs and growth of all tumours including lymph node metastases and microtumours was inhibited. In every tumour, tumour blood flow began to decrease immediately after AC7700 administration and reached a minimum at approximately 30 min after injection. In many tumour capillaries, blood flow completely stopped within 3 min after AC7700 administration. These results demonstrate that AC7700 is effective for tumours growing in various tissues and organs and for metastases. We conclude that tumour blood flow stanching induced by AC7700 may become an effective therapeutic strategy for all cancers, including refractory cancers because the therapeutic effect is independent of tumour site and specific type of cancer.

*British Journal of Cancer* (2002) **86**, 1604–1614. DOI: 10.1038/sj/bjc/6600296 www.bjcancer.com

© 2002 Cancer Research UK

## 

The ultimate goal of cancer chemotherapy is the attack and elimination of all cancer cells that develop in the body. The target of anticancer drugs has thus inevitably become the cancer cell. However, a chemical substance that shows strong cytotoxicity against cancer cells has similar effects against normal cells that are actively dividing, such as haemopoietic cells and alimentary canal mucosa cells, resulting in severe side effects. At present, very few drugs exhibit tumour-selective toxicity.

Many researchers have considered the possibility that tumour growth might be suppressed by cutting off the supply of nutrients to tumours. Two approaches are currently being studied to accomplish this. One is an antiangiogenic approach, which was first proposed by Folkman (1971). Folkman postulated that tumour growth depends on angiogenesis and that if tumour angiogenesis could be inhibited, an effective therapeutic strategy could be developed for solid tumours. This approach stresses inhibition of tumour vascularisation, which is the lifeline for supply of nutrients to tumours. To date, a number of antiangiogenic compounds have been screened, and several are currently in clinical trials as candidate antiangiogenic drugs (Kerbel, 2000; Liekens *et al*, 2001).

The second approach was first suggested by Algire *et al* (1954). A strategic point in this approach is to shut off the tumour blood flow (TBF) into tumour tissue and thus stop the tumour's nutritional supply. Algire *et al* (1954) found out that podophyllotoxin completely shut off the TBF. Podophyllotoxin was not applied clinically, however, because of its strong toxicity. A research group in the United Kingdom and New Zealand continued to develop this approach and found that drugs related to flavone acetic acid and tubulin-binding agents such as vinca alkaloids markedly decreased TBF (Bibby *et al*, 1989; Hill *et al*, 1989, 1993; Zwi *et al*, 1989; Baguley *et al*, 1991; Ching *et al*, 1994; Chaplin *et al*, 1996; Holwell *et al*, 2001). However, the decrease in TBF by flavone acetic acid was accompanied by a marked reduction in systemic blood pressure, and an effective decrease in TBF by vinca alkaloids was achieved only at doses approaching the maximum tolerated dose.

In 1989, Pettit *et al* (1989) isolated a novel compound from the African shrub *Combretum caffrum* and named it combretastatin A-4 (CS A-4). This compound was found to have potent inhibitory activity against tubulin polymerisation. Subsequently, the water-soluble prodrug combretastatin A-4 phosphate (CS A-4-P) was synthesised for *in vivo* testing. Dark *et al* (1997) first demonstrated that systemic administration of the prodrug induced a marked decrease in vascular perfusion in experimental and human breast cancer models *in vivo*. Several recent studies reported that CS A-4-P showed antitumour effects against various kinds of tumours without severe side effects (Horsman *et al*, 1998; Chaplin *et al*, 1999; Grosios *et al*, 1999; Tozer *et al*, 1999; Landuyt *et al*, 2000).

Another novel combretastatin derivative, AC7700, was synthesised in Japan (Hatanaka *et al*, 1998; Ohsumi *et al*, 1998a,b). For colon 26 adenocarcinoma, AC7700 showed stronger antitumour effects, compared with CS A-4-P, at a dose that was an order of magnitude lower than that of CS A-4-P (Nihei *et al*, 1999a). Our research group also demonstrated that AC7700 has a remarkable therapeutic effect against not only transplanted tumours (Hori *et al*, 1999; Nihei *et al*, 1999b) but also methylcholanthrene-induced autochthonous primary tumours (Hori *et al*, 2001), and we determined that the effect is caused by irreversible stoppage of TBF (Hori *et al*, 1999).

Most investigations of the correlation between TBF and antitumour effects have been performed with subcutaneous tumours. Before clinical trials are undertaken, however, comprehensive research is needed to determine whether AC7700 can stop the TBF not only of subcutaneous tumours but also of tumours developing in various tissues and organs, and whether it is capable of inhibiting the growth of systemically disseminated metastatic tumours. This research is required because cancer chemotherapy is usually used for patients with tumours in various internal organs and especially patients with metastatic lesions. For such research, therefore, we have produced solid tumours growing in the liver, stomach, kidney, muscle tissue, and lymph nodes by implanting tumour cells into those tissues.

The purpose of the present study was to investigate whether AC7700 stops the TBF of tumours growing in various tissues and organs, including lymph nodes, and whether AC7700 inhibits the growth of tumours disseminated systemically. On the basis of our results, we discuss the possibility that long-lasting stoppage of TBF may become a novel therapeutic strategy against refractory types of cancer as well as various kinds of solid tumours.

## MATERIALS AND METHODS

### Rats and tumour

Male Donryu rats (Crj-Donryu; Nippon Charles-River, Yokohama, Japan), 8–10 weeks old and with an average weight of 250–300 g, were used for TBF measurements and vital microscopic observations. Rats of the same strain, weighing 200–220 g each, were used for the therapeutic experiment. Rats were bred and maintained in a ventilated, temperature-controlled (24±1°C), specific pathogen-free environment, on a bed of wood shavings, with food and water freely available and a 12 h light–dark cycle. They were usually housed two or three per cage. The rats were fitted with transparent chambers for microscopic observations (see below) and were caged singly.

Tumour cells that were used included LY80, a variant of the Yoshida sarcoma, and Sato lung carcinoma (SLC), an undifferentiated lung carcinoma. In our laboratory, LY80 and SLC are maintained by successive i.p. and s.c. transplantations, respectively. We chose SLC for vital microscopic observations because when the tumour is implanted in transparent chambers, demarcation between the edge of the growing tumour and normal tissue is clear, and therefore the change in microtumour size after therapy can easily be measured (Hori *et al*, 1995). Experiments were performed with the animals anaesthetised in a controlled-temperature box fitted with a suction duct. All experimental protocols were reviewed by the Committee on the Ethics of Animal Experiments in our institute and were carried out in accordance with Guidelines for Animal Experiments issued by Tohoku University School of Medicine and The Law (No. 105) and Notification (No. 6) issued by the Japanese Government. The ethical guidelines that were followed meet the standards required by the UKCCCR (1998) guidelines.

### Tumour cell implantation into various tissues

LY80 cells growing in ascites of a donor rat were collected, suspended in phosphate-buffered saline, and adjusted to a concentration of 2×10^6^ cells per 10 μl. Recipient rats were anaesthetized with diethyl ether (Wako Pure Chemical Industries, Ltd., Osaka, Japan). For implantation of tumour cells in the liver or stomach, a midline incision 1.5–2 cm long was made. The tumour cell suspension was drawn into a 50-μl graduated syringe (Hamilton Co., Reno, NV, USA), and a 27-gauge syringe needle (27G; Termo Co., Tokyo, Japan) was used to inject 10 μl directly into the left lobe of the liver or into the muscularis propria in the stomach. Injection depths were 2 and 1 mm, respectively, from the surface of the liver and stomach. The needle holes were sealed with synthetic resin glue (Aron Alpha 201; Toagosei Chemical Industry Co., Tokyo, Japan). The incision wound was closed, and the animals were allowed to recover. For tumour cell implantation into the kidney, rats were anaesthesized and placed in the left lateral decubitus position, and a vertical incision was made in the right flank through the skin and peritoneum to expose the lateral aspect of the kidney. The tumour cells (2×10^6^ cells in 10 μl) were implanted into the renal parenchyma by use of the above-mentioned syringe and needle. The site of injection was 2 mm below the renal capsule. After injection, the operation wound was closed in one layer with thread. For tumours growing in muscle, tumour cells (2×10^6^ cells in 0.1 ml) were injected into the left biceps femoris as described for the kidney.

### Lymph node metastasis

Lymph node metastasis was induced according to the method reported previously (Hori *et al*, 1979). That is, the tumour cell suspension (2×10^6^ cells in 10 μl) was slowly injected into the middle of the ear by using a 27-gauge syringe needle. First, a tumour grew only at the site of the inoculation. When the largest diameter of the tumour was approximately 7 mm, primary tumours were removed by cutting off the ear. After a further 7–10 days, metastatic foci were formed in the anterior or posterior cervical lymph node.

### Drugs

AC7700 ((Z)-N-[2-methoxy-5-[2-(3,4,5-trimethoxyphenyl) vinyl] phenyl]-L-serinamide hydrochloride), one of the combretastatin derivatives, was synthesised and provided by Ajinomoto Pharmaceutical Research Laboratories, Kawasaki, Japan. The intensity of TBF stanching or antitumour activity was increased by replacing an OH group of the B ring of CS A-4 with an NH_2_ group. In addition, water solubility of the compound was markedly improved by substituting serinamide for the NH_2_ group. Properties of this compound have been reported elsewhere (Hatanaka *et al*, 1998; Ohsumi *et al*, 1998a,b). The AC7700 powder was dissolved in 0.9% NaCl solution, to give a final concentration of 10 mg ml^−1^, immediately before use. The solution (10 mg kg^−1^) was injected into the tail vein at a rate of 0.15 ml min^−1^ by using an infusion pump (Compact Syringe Pump; Harvard Apparatus Co., Inc., Millis, MA, USA). Pentobarbital sodium (Nembutal; Abbott Laboratories, North Chicago, IL, USA) and enflurane (Ethrane; Abbott Laboratories) were used for anaesthesia. Pentobarbital was administered i.m. at a dose of 25 mg kg^−1^ 10 min before the experiment, and supplemental doses (12.5 mg kg^−1^ i.m.) were given at 90-min intervals to maintain immobilization during the experiment. Enflurane concentration was maintained at 1% in the inhaled carrier gas at 1 l min^−1^ by means of an anaesthetic apparatus for small laboratory animals (Hori *et al*, 1991).

### Measurements of mean arterial blood pressure (MABP)

MABP was monitored in all rats in which TBF was measured. MABP was measured via a catheter (PE-50; Clay Adams, Persippany, NJ, USA) inserted into the right femoral artery. Pressure in the catheter was recorded with a pressure transducer (TNF-R; Spectramed Medical Products, Singapore), the output of which was fed into an amplifier (6M82; NEC-Sanei Co., Tokyo, Japan) adapted for MABP measurement.

### Measurement of TBF

TBF was measured with the hydrogen clearance method (Hori *et al*, 1991, 1996) whose principle was described in detail by Aukland *et al* (1964). In brief, after saturation of the tissue with hydrogen following inhalation of 9% hydrogen gas in air (at 1 l min^−1^), the blood flow value (in ml min^−1^ 100 g^−1^ tissue) was calculated from the half-life of the clearance curve obtained. A tissue blood flow meter with two separate amplifiers (PHG-201; Unique Medical Co., Tokyo, Japan) was used. Two hydrogen electrodes with 80-μm diameters (UHE-201C; Unique Medical) and two rod-type Ag/AgCl reference electrodes (TT-98012; Unique Medical), which were inserted between the skin and musculature in the caudal region, were used for each rat. In some rats with tumours growing in the kidney or liver, one electrode was inserted in the tumour and the other electrode was inserted in the kidney cortex or liver within 3 mm of the tumour nodule, for simultaneous measurement of blood flow changes in both the tumour and the adjacent normal tissue.

Blood flow measurements in tumours implanted in the liver, stomach, kidney, or muscle were performed 7–10 days postinoculation. To measure the TBF of tumours growing in the liver or stomach, laparotomy was performed at the same abdominal wall as that used for the earlier incision, and electrodes were inserted into the tumour. Electrodes were inserted to depths of 3 and 2 mm from the surface of the liver tumour and the stomach tumour, respectively. For measurements of the TBF in the kidney tumour, a vertical incision was carefully made in the right flank through the skin and peritoneum, similar to the incision for implantation of the tumour, and electrodes were inserted into the solid tumour growing in the renal parenchyma. Electrodes were inserted to depths of 2 mm from the tumour surface. The TBF in the intramuscular tumours was measured by introducing electrodes to a depth of 3 mm below the tumour surface. For measurements of TBF in lymph node metastasis, a syringe needle (23G; Termo Co.) was used to make a pinhole in the skin overlying a tumour growing in the cervical lymph node, and a hydrogen electrode was inserted through the hole into the tumour tissue. The electrode was inserted to a depth of 3 mm from the surface of the skin. For TBF measurements of tumours in the kidney, muscle or lymph nodes, rats were placed prone on a heated stage at 34°C. For tumours in the liver or stomach, rats were placed in a supine position.

Throughout the experiment, the animals were kept in the same position, and rectal temperature was monitored with a thermistor for small animals (PTC-201; Unique Medical) and was maintained at 33.5–35.5°C. All rats were killed with ether at the end of the experiment. A check was performed to determine whether electrodes were located in the tumours.

### Change in TBF caused by AC7700 or 0.9% NaCl solution

By means of the same method as described previously (Hori *et al*, 1999), the TBF in tumours growing within the liver, stomach, kidney, muscle, and lymph nodes was measured at multiple time points (i.e., 10, 30, and 60 min, and every subsequent hour) until 6 h after AC7700 or 0.9% NaCl solution administration.

### Rat transparent chamber and vital microscopic observation of changes in tumour and TBF caused by AC7700

Rat transparent chambers (Hori *et al*, 1990) were implanted in dorsal skin flaps under aseptic conditions. Each chamber consisted of two identical titanium frames containing a circular quartz glass window 300 μm thick. After anaesthesia, a rat with a transparent chamber was placed in the right lateral position on a heated stage (MATS-SFA; Tokai HIT Co., Ltd., Tokyo, Japan), at 34.5°C, which was attached to the mechanical stage of the microscope.

AC7700 (10 mg kg^−1^) was administered via the lateral tail vein by using an infusion pump. The change in TBF and the tumour degradation process were directly observed via a light microscope (Eclipse E800; Nikon Corporation, Tokyo, Japan) with a 10× ocular (CFI UW; Nikon) and 2–20× objectives (CFI Plan Flour; Nikon). Tumour vessels within the chamber were transluminated by a 12-V 100-W halogen lamp. The microscopic image was recorded using a closed-circuit video system consisting of a CCD video camera (CS-900; Olympus Kogaku K.K., Tokyo, Japan), a TV monitor (PVM-14M4J; Sony Corporation, Tokyo, Japan), and a S-VHS video recorder (SVO-2100; Sony). A video timer was superimposed on images for record keeping. Segments of the video tape containing desired images were transferred to computer hard disk (Power Macintosh 8600/200, Apple Japan, Inc., Tokyo, Japan). Final images were output by a digital printer (Pictography 4000, Fuji Photo Film Co., Ltd., Tokyo, Japan).

### Chemotherapy against tumours growing in various organs

As a model for systemic metastases, LY80 cells (2×10^6^ cells in 0.1 ml of phosphate-buffered saline) were injected into 16 rats via the lateral tail vein at a rate of 0.15 ml min^−1^ using an infusion pump (Compact Syringe Pump). Tumour-bearing rats were divided into two groups: group I, to which the 0.9% NaCl solution was administered (eight rats); and group II, to which AC7700 at 10 mg kg^−1^ was administered (eight rats). Treatments were performed five times at intervals of 2 days, starting on day 7 after tumour cell injection. All rats were killed with ether and examined macroscopically 2 days after the last treatment. For microscopic examinations, brain, lung, heart, liver, spleen, kidney, gastrointestinal tract, and tumours were resected and fixed in 15% formalin, processed, and embedded in paraffin.

#### (a) Therapeutic effect of AC7700 against tumours growing in lung, liver, heart, and subcutis

In a preliminary experiment, we had confirmed that on the seventh day after i.v. transplantation of LY80, all rats developed tumours in the lung, liver, heart, subcutis, and visceral lymph nodes. A prominent characteristic of LY80 cells was that they lodged in the myocardium and grew there. In the present experiment, the therapeutic effect of AC7700 was judged according to the difference in the number and size of tumours growing in the lung, heart, liver, and subcutis between the AC7700-treated group and the 0.9% NaCl-treated group. The occurrence of fluid in the thoracic cavity was also checked. Tumours were counted in the maximum section of the left lung, myocardium of the left ventricle, and left lobe of the liver. Each section was projected with a profile projector (V-16; Nikon) and tumour regions were traced. The tumour area was calculated by using an area analyser (WT-4400SE; WACOM Co., Saitama, Japan). The weights of the skin tumours were measured with an electronic balance (JP-3000WP; Chyo Balance Co., Kyoto, Japan).

#### (b) Therapeutic effect of AC7700 against lymph node metastases

The therapeutic effect of AC7700 on lymph node metastases was evaluated by use of the difference in the number and weight of lymph node metastases between the AC7700-treated group and the 0.9% NaCl-treated group. All tumours visible to the naked eye in the mediastinal, gastrointestinal, lumbar, adrenal, axillary, and inguinal lymph nodes were removed, and their weights were measured.

#### (c) Therapeutic effect of AC7700 against a microtumour growing in the transparent chamber

This therapeutic effect was evaluated by assessing the difference in the growth curve between the AC7700-treated group and the 0.9% NaCl-treated group. Photographs of the tumour were taken before and 24 and 48 h after drug administration. A transparent vinyl sheet was placed over the photograph, and the tumour was traced onto the overlay. Tumour area was measured by an area analyser (WT-4400SE) and was expressed as a percentage compared with the area at 0 h.

### Statistics

Results were expressed as means±s.d. The statistical significance of TBF reduction at each time point after AC7700 administration was evaluated with repeated measures ANOVA. Comparisons of tumour volume, weight, and number and of the body weights of the animals were made using Mann–Whitney *U*-tests. *P* values of 0.05 or lower were considered significant.

## RESULTS

### Effect of AC7700 on TBF in tumours growing in the liver, stomach, kidney, and muscle, and in lymph node metastases

**Figure 1:**
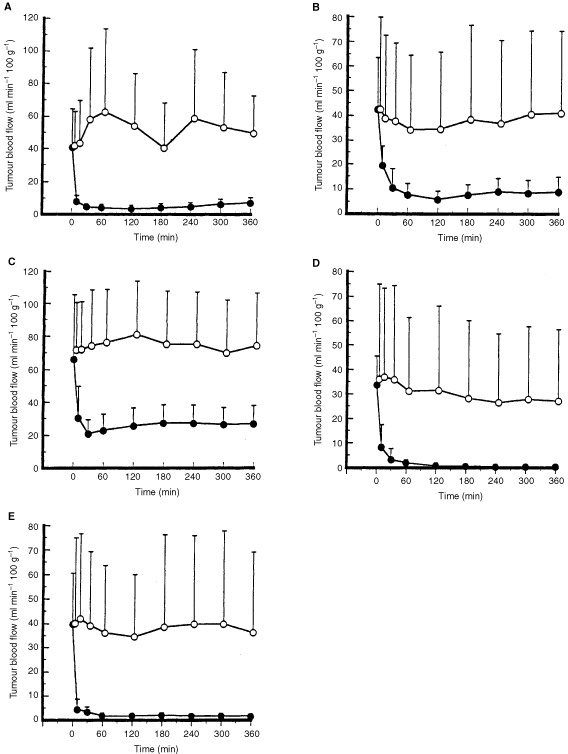
Tumour blood flow changes caused by AC7700 in tumours growing in various tissues and organs. AC7700 at 10 mg kg^−1^ or 0.9% NaCl solution was infused via the lateral tail vein at the rate of 0.15 ml min^−1^ by an infusion pump at 0 min. The TBF significantly decreased in all tumours after AC7700 administration compared with the control group. The TBF of tumours in the liver, muscle, and lymph node was completely stopped 30–60 min after AC7700 administration. (**A**) tumour growing in the liver (open circle, 0.9% NaCl solution (*n*=8) *vs* solid circle, 10 mg kg^−1^ AC7700 (*n*=10), *P*=0.0007); (**B**) tumour growing in the muscularis propria of the stomach (open circle, 0.9% NaCl solution (*n*=8) *vs* solid circle, 10 mg kg^−1^ AC7700 (*n*=14), *P*=0.0118); (**C**) tumour growing in the kidney (open circle, 0.9% NaCl solution (*n*=8) *vs* solid circle, 10 mg kg^−1^ AC7700 (*n*=10), *P*=0.0004); (**D**) tumour growing in the muscle (open circle, 0.9% NaCl solution (*n*=8) *vs* solid circle, 10 mg kg^−1^ AC7700 (*n*=10), *P*=0.0243); (**E**) metastatic foci in the cervical lymph node (open circle, 0.9% NaCl solution (*n*=8) *vs* solid circle, 10 mg kg^−1^ AC7700 (*n*=10), *P*=0.0065).

Tumour blood flow changes in tumours growing in the liver, stomach, kidney, and muscle and in lymph node metastases in response to i.v. administration of 10 mg kg^−1^ AC7700 or 0.9% NaCl solution are shown in Figure 1

. Tumour blood flow reduction was highly significant in all tumours in the AC7700-treated group compared with the 0.9% NaCl-treated group. In the case of tumours in the liver, muscle and lymph nodes, TBF was completely stopped within 1 h. When blood flow changes in the normal liver and the hepatic tumour were measured simultaneously, TBF was almost completely stanched by AC7700, whereas the blood flow decrease in the normal liver was 30–40% at the maximum and was reversible (data not shown). The rate of decrease and the pattern of recovery of liver blood flow were almost the same as those reported previously (Hori *et al*, 1999).

**Figure 2:**
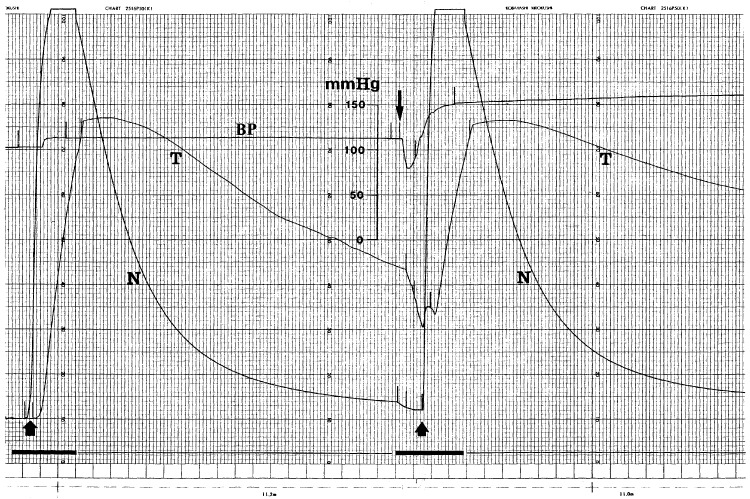
Representative tracings of MABP and the hydrogen gas clearance curve. Upward arrow, start of 9% H_2_ inhalation; N, clearance curve in kidney cortex (normal tissue); T, clearance curve in tumour growing in the kidney; downward arrow, starting point of i.v. injection of 10 mg kg^−1^ ml^−1^ AC7700; the bold line at the bottom, chart speed 3 cm min^−1^; the thin line at the bottom, chart speed 60 mm min^−1^. Tissue blood flow was calculated from the half-life of H_2_ washout. Before AC7700 administration, tissue blood flow in the normal kidney cortex and tumour was 157.5 and 30.9 ml min^−1^ 100 g^−1^, respectively. After AC7700 was administered i.v., MABP transiently decreased from 113 to 80 mmHg and then immediately increased to 157 mmHg. Under AC7700-induced hypertension, although tissue blood flow in the kidney cortex showed almost no change (150.7 ml min^−1^ 100 g^−1^), the TBF decreased to 13.8 ml min^−1^ 100 g^−1^.

The TBF decrease caused by AC7700 in the renal tumour was completely tumour specific. Typical changes in clearance curves for the renal cortex and renal tumour after AC7700 administration are shown in Figure 2

. In this case, although the MABP decreased transiently from 113 to 80 mmHg after i.v. administration of 10 mg kg^−1^ AC7700, it then began to increase and reached 157 mmHg. Meanwhile, TBF in the renal tumour decreased from 30.9 to 13.8 ml min^−1^ 100 g^−1^ within 3 min. Blood flow in the renal cortex, however, was 157.5 and 150.7 ml min^−1^ 100 g^−1^ before and after AC7700 administration, respectively. Thereafter, TBF continued to decrease, and it was approximately 8 ml min^−1^ 100 g^−1^ after 6 h. However, renal cortex blood flow remained approximately 150 ml min^−1^ 100 g^−1^ throughout the experiment.

The effect of AC7700 on TBF in lymph node metastases was independent of tumour size, and the TBF of all tumours, including microfoci with a diameter of 4 mm or less, was completely stopped.

**Figure 3:**
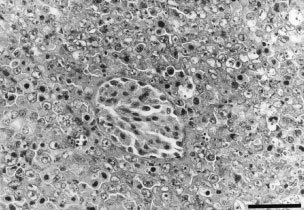
Photomicrograph of a measured region in a renal tumour. A typical histological feature is present: glomerulus survives within the tumour. H&E-stained tissue section. Original magnification, ×400. Bar, 50 μm.

In tumours within muscle, liver, and lymph nodes, no preexisting tissues remained within the regions measured. In particular, in lymph node metastases, even in very small growing foci (less than 4 mm in diameter), preexisting tissues were not observed (data not shown). In contrast, in renal tumours, even in tumours with a diameter greater than 8 mm, glomeruli survived within regions assayed (Figure 3

).

### Antitumour effect of AC7700 against tumours growing in various tissues and organs

**Figure 4:**
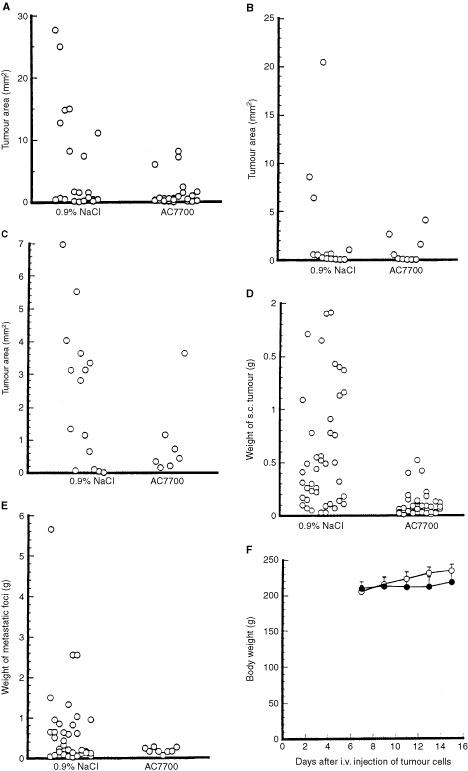
Antitumour effect of AC7700 against tumours growing in various tissues. The left side in each panel shows results for the group given 0.9% NaCl (eight rats); the right side in each panel shows results for the group treated with 10 mg kg^−1^ AC7700 (eight rats). The sum of tumours that appeared in rats in each group is shown in each panel. (**A**) tumours in the lung; (**B**) tumours in the liver; (**C**) tumours in the myocardium; (**D**) tumours in the subcutis; (**E**) lymph node metastases (mediastinale, coeliacum, mesentericum, lumbare); (**F**) changes in body weight of tumour-bearing rats during AC7700 treatment. (**A**–**C**), mean tumour area±s.d. (mm^2^) in the maximum sections of the left lung, the left lobe of the liver, and the myocardium of the left ventricle. (**D**) and (**E**) mean tumour weight±s.d. (g) in the subcutis and lymph node metastases. Tumour size in the lung and subcutis in the AC7700-treated group was significantly smaller than that in the 0.9% NaCl-treated group (lung tumour, *P*=0.0228; subcutaneous tumour, *P*<0.0001). The number of lymph node metastases was significantly smaller in the AC7700-treated group compared with the control group (*P*=0.0054). There was no significant difference in body weight between the two groups.

Effects of AC7700 on growth of tumours developing in the lung, liver, heart, subcutis, and visceral lymph nodes are shown in Figure 4A

–E. The size of tumours in the lung and subcutis and the number of lymph node metastases were significantly suppressed in the treatment group compared with the control group. Growth of the tumours in the liver and heart tended to be inhibited by AC7700. Haemothorax was observed in four of eight rats in the control group, and those rats had large tumours in the lung and the mediastinal lymph node. In the AC7700-treated group, in contrast, the growth of those tumours was markedly suppressed, and, therefore, no rats had haemothorax. There was no significant difference in body weight between the AC7700-treated group and the control group (*P*=0.1069) (Figure 4F). No obvious side effects, such as anaemia or diarrhoea, were observed at the AC7700 dose used in the protocol of this treatment. This experiment was repeated twice using the same protocol, with similar results.

### Vital microscopic observation of TBF change and therapeutic effect caused by AC7700

**Figure 5:**
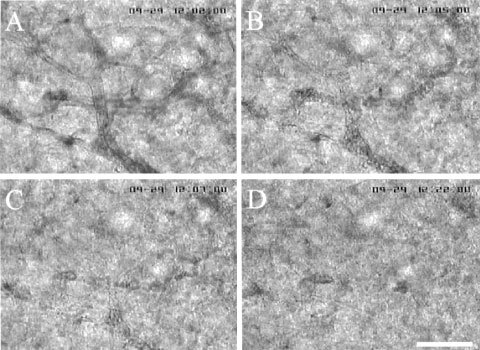
Blood flow stasis in tumour capillaries caused by 10 mg kg^−1^ AC7700. (**A**) before AC7700 administration; (**B**) 3 min later; (**C**) 5 min later; (**D**) 20 min later. Tumour blood flow in this region completely stopped approximately 2 min after the AC7700 administration. Original magnification, ×100. Bar, 100 μm.

Tumour blood flow always began to decrease immediately after AC7700 administration and completely stopped within 30–60 min. An example of AC7700-induced TBF cessation in a tumour microcapillary is shown in Figure 5

**Figure 6:**
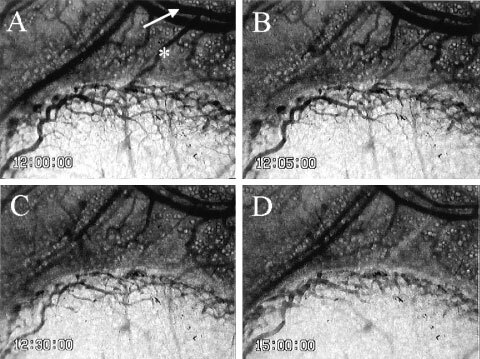
Contraction of a host arteriole and a tumour-feeding arteriole caused by 10 mg kg^−1^ AC7700. (**A**) before AC7700 administration; (**B**) 5 min later; (**C**) 30 min later; (**D**) 3 h later. Tumour blood flow completely stopped 30 min after i.v. administration of AC7700. Note that an arteriole (white arrow) underwent marked contraction in response to AC7700, and a feeding arteriole (asterisk) into a tumour disappeared from view. Original magnification, ×20. Bar, 250 μm.

. In this case, TBF completely stopped within 3 min after i.v. injection of 10 mg kg^−1^ AC7700. Arterioles in normal tissue and arterioles feeding into tumours markedly contracted after AC7700 administration (Figure 6

). During this continuous arteriolar contraction, the TBF completely stopped; the MABP rose simultaneously with the contraction of these arterioles.

Figure 7

**Figure 7:**
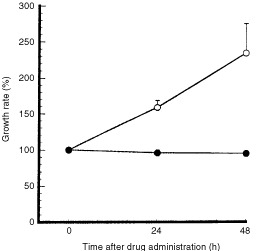
Inhibition of SLC tumour growth caused by AC7700 in the tumour developing in a transparent chamber. Tumour size at the start of observation was defined as 100%. During the 48 h after a single i.v. administration of 10 mg kg^−1^ AC7700, tumour size did not change at all (solid circle) (*n*=4). In contrast, tumours in the control group continued to grow (open circle) (*n*=4). Tumour area doubling time was 41.7±11.4 h.

shows the effect of a single i.v. injection of 10 mg kg^−1^ AC7700 or 0.9% NaCl solution on the size of a microtumour growing in the transparent chamber. The area doubling time of the SLC tumour was 41.7±11.4 h (*n*=4) in the control group. However, tumours completely stopped growing during 48 h of observation after administration of AC7700 (*n*=4). One example of the therapeutic effect of AC7700 against a small SLC tumour with a diameter of less than 3 mm is shown in Figure 8

**Figure 8:**
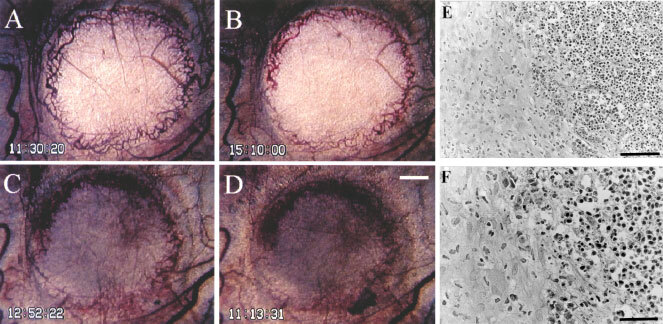
Typical finding of growth inhibition caused by AC7700 in an SLC microtumour developing in a transparent chamber. (**A**) before administration of 10 mg kg^−1^ AC7700 administration; (**B**) 3.5 h after administration of AC7700; (**C**) 25 h later; (**D**) 48 h later; (**E**) histology 48 h later. Tumour blood flow completely stopped at 1 h after a single i.v. administration of AC7700. The whole region of the tumour, with a diameter of 2.5 mm, became necrotic. Tumours stopped growing completely during the 48-h observation period. Histological study (**E** and **F**, H&E stained) certified the tumour (shown on the right side) as necrotic. Original magnification: **A**–**D**, ×20; **E**, ×200; **F**, ×400. Bars: **A**–**D**, 500 μm; **E**, 100 μm; **F**, 50 μm.

**Figure 9:**
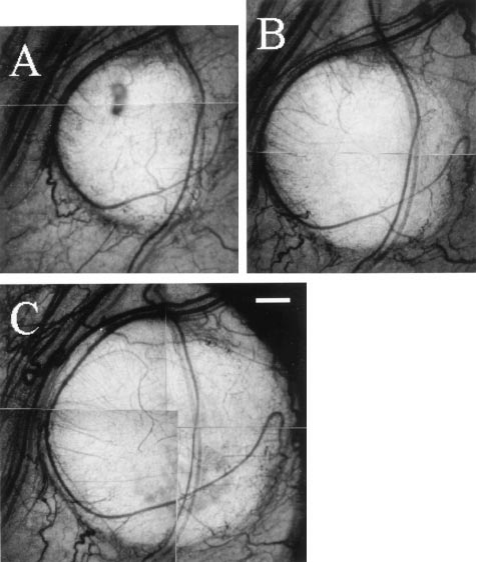
Typical finding of growth of an SLC tumour developing in a transparent chamber in the control group. (**A**) 0 h; (**B**) 24 h later; (**C**) 48 h later. The tumour never stopped growing during the observation period. Original magnification, ×20. Bar, 500 μm.

. Tumour blood flow in the whole area of the tumour was completely shut off, and the colour of the tumour changed from white to brown within 24 h after administration of AC7700 (Figure 8A–D). Histological examination 48 h later confirmed that this tumour had become necrotic (Figure 8E, F). In contrast, the colour of the tumour in the control group did not change at all, and the tumour continued to grow during the observation period (Figure 9

).

## DISCUSSION

The present study clearly demonstrated that i.v. administration of AC7700 strongly stanched TBF not only in subcutaneous tumours (Hori *et al*, 1999, 2001) but also in tumours growing in various kinds of tissues and organs, including lymph node metastases, and that the stanched TBF suppressed tumour growth in all regions of the body. In all tumours, AC7700-induced TBF cessation was completed within 30–60 min after i.v. administration of the drug and was maintained during the experimental period of 6 h. This result was analogous to that obtained with subcutaneous tumours (Hori *et al*, 1999). In tumours growing in muscle, liver, and lymph nodes, the TBF decreased almost to zero. In particular, the TBF in the lymph node was completely shut off, even in small metastases with diameters less than 4 mm, whose initial blood flow was often higher than 80 ml min^−1^ 100 g^−1^. This is a noteworthy phenomenon that could be exploited as a therapeutic strategy against micrometastases in lymph nodes.

Denekamp *et al* (1983) investigated the effect of TBF on tumour growth by mechanically stopping the TBF and found that if stoppage was continued for more than 18 h, tumours never showed renewed blood circulation and lost the ability to grow. We reported in a previous paper (Hori *et al*, 1999) that once the TBF was stopped by AC7700, it did not recover within tumours, even 24 h after drug administration in many regions. The present study shows that the more complete the TBF stanching, the stronger the inhibitory effect against tumour growth. In fact, for even a small tumour with a diameter of less than 3 mm growing in a transparent chamber, if the TBF cessation was complete and long-lasting, the whole region, including the peripheral rim of the tumour, became necrotic and tumour growth eventually stopped. In the AC7700-treated group, the reduced number of lymph node metastases was certainly caused by complete TBF stanching in these metastases.

Simultaneous measurement of blood flow in the renal cortex and the renal tumour showed that the TBF decreased markedly as a result of AC7700, but blood flow in the renal cortex hardly changed, even in regions only 3 mm from the tumour edge. However, TBF stanching in renal tumours was not as complete as that in tumours growing in other organs. The reason for this difference is probably related to the fact that many glomeruli, which naturally have high blood flow, sporadically survived in measured tumours.

We previously showed (Hori *et al*, 1999) that although the blood flow in normal tissues tended to decrease at the dose of AC7700 used in this study, it was never shut off. The decrease was comparatively small and reversible in most cases. We consider this to be the reason that AC7700 can induce selective tumour necrosis without causing severe damage to normal tissues or organs. Although the degree of blood flow decrease in normal tissues was not as large as that in tumours, except in bone marrow (Hori *et al*, 1999), in a clinical trial the dose of AC7700 should be carefully chosen, because in older patients with compromised perfusion in organs such as heart and brain, a slight reduction in perfusion may have serious consequences.

It has not yet been sufficiently clarified why AC7700 causes TBF to stop and why the TBF stoppage is irreversible, whereas the decrease in blood flow in normal tissues is reversible. Some authors speculate that TBF stanching due to a combretastatin compound may be caused by a direct effect of the drug on tumour vessel endothelial cells (Dark *et al*, 1997; Grosios *et al*, 1999). However, *in vivo* evidence for this suggestion has not yet been provided. Although it is not clear whether TBF stanching is caused by direct action of this compound on tumour vessels, it seems quite likely that some structural change would occur in tumour vessels in any site after AC7700 administration.

Tumour vessels, even large-calibre vessels, usually consist of only a monolayer of endothelial cells and have no pericytes or smooth muscle cells for a supporting structure; sometimes a basement membrane cannot be observed at all or only incompletely (Papadimitriou and Woods, 1975; Konerding *et al*, 1989). Normal vessels, in contrast, are usually supported by pericytes and a continuous basement membrane (Simionescu and Simionescu, 1984). The difference in blood flow change between normal and tumour tissues may be related to this different vessel architecture. In fact, we observed that lumens of many tumour vessels that had rich blood flow and thus a large volume at the time of AC7700 administration completely flattened after the TBF was shut off; normal capillaries did not flatten out, even after blood was no longer present in vessels, and lumen morphology was retained (in preparation for submitting). We are now obtaining data suggesting that strong and long-lasting vasoconstriction of arterioles and tumour-feeding arterioles leads to irreversible TBF stoppage and thus extensive tumour cell death. We will describe in detail the microvascular mechanism of TBF stanching caused by AC7700 in a separate paper.

In summary, AC7700 stopped TBF at a dose that did not cause severe side effects regardless of the growing site of the tumour and markedly suppressed tumour growth. The efficacy of cancer chemotherapy based on TBF stanching is in theory independent of the type of cancer cell. Accordingly, this strategy may become a powerful therapeutic tool against refractory cancers, and AC7700 is a promising anticancer compound that shows such effects.
